# Effectiveness of nurse-run smoking cessation discussions lasting up to 90 minutes: A systematic review and meta-analysis

**DOI:** 10.18332/tpc/215042

**Published:** 2026-02-21

**Authors:** Klas Winell, Juha E. Ahonen

**Affiliations:** 1 Conmedic, Espoo, Finland; 2 Department of Public Health, University of Turku, Turku, Finland; 3 The Finnish Medical Society Duodecim, Helsinki, Finland

**Keywords:** smoking, cessation, counselling, nurse, health promotion

## Abstract

**INTRODUCTION:**

It is important that nurses are active in smoking cessation. We studied if cessation discussions by nurses, lasting up to 90 minutes, lead to cessation of smoking.

**METHODS:**

A literature search was made from The Cochrane Central Register of Controlled Trials and MEDLINE. Randomized controlled trials on smoking cessation of adult daily smokers by nurses published from January 1983 to December 2023 were searched with the following defining of intervention: at least once face-to-face contact, a maximum of five contacts, total counselling time of ≤90 minutes, no concurrent cessation medication or physician input. Controls had no nurse counselling. The restricted maximum-likelihood method was used to calculate the odds ratio (OR) without adjustment for individual trials and to estimate the pooled effect. RoB 2 tool was used to assess the bias.

**RESULTS:**

Seven studies involving 4443 smokers were included. All studies presented some risk of bias, and three were judged to have high overall risk of bias. The pooled analyses favored the controls but without a statistically significant effect at 12 months, biochemically validated abstinence (4295 smokers, six studies, OR=1.22; 95% CI: 0.83–1.80) and self-reported abstinence (3396 smokers, five studies, OR=1.02; 95% CI: 0.65–1.61). Substantial heterogeneity was observed (I^2^=57% for biochemically validated outcomes; I^2^=73% for self-reported outcomes).

**CONCLUSIONS:**

Our results of studies on nurse-run counselling lasting ≤90 minutes did not demonstrate effectiveness in promoting smoking cessation. This should be considered while organizing cessation care. Instead, smokers who are interested in using cessation medication should be given more time and offered the possibility to participate in cessation group counselling. Given that many participants in the included studies had long smoking histories and smoking-related illnesses, future RCTs should examine nurse-run cessation interventions in populations with shorter smoking histories and fewer comorbidities.

## INTRODUCTION

Smoking continues to be a major global health concern, contributing to high rates of morbidity and mortality^1^. Although smoking prevalence has declined in many regions, including by approximately 10 percentage points in Europe and the United States since 2000^2^, tobacco use remains a leading cause of preventable disease and death. Public health strategies including stronger regulations to prevent youth initiation^3^, increased taxation^4^, and activity of healthcare personnel to prevent smoking and support cessation remain essential.

Nurses play an important role in health promotion and are increasingly involved in task-sharing interventions that were traditionally physician-run. Nurse-run interventions have shown benefits in chronic disease management, including improving diabetes care^5^, lowering low-density lipoprotein cholesterol^6^, reducing hypertension^7^, and decreasing complications associated with atrial fibrillation^8^. Previous evidence has suggested that nurse-run smoking cessation interventions can be effective. A Cochrane review from 2017 reported moderate-quality evidence that nurse-delivered cessation support increased smoking cessation (risk ratio, RR=1.29; 95% CI: 1.21–1.38)^9^. However, these interventions varied considerably in intensity and duration.

We believe that nurses could take greater responsibility for smoking cessation counselling because they already do a lot of life-style promotion with patients. To encourage the transfer to nurses one needs solid evidence that it is beneficial and, even better, is cost-effective.

Because counselling time in clinical practice is often limited, it is important to determine whether brief nurse-run smoking cessation discussions are effective. This systematic review and meta-analysis aimed to evaluate the effectiveness of such interventions in adult smokers, compared with no intervention or brief usual care.

## METHODS

### Eligibility criteria

We included randomized controlled trials (RCTs) of nurse-run smoking cessation interventions that met the following criteria.

### Population

Adults (≥18 years) who were daily smokers or used e-cigarettes. They had smoked at least one cigarette a day before entering the study or in case the recruitment to the study took place at hospital they had to be smoking before the hospital episode.

### Intervention

At least one face-to-face counselling session with a nurse, with no physician involvement other than referral; maximum five contacts (face-to-face or remote); total counselling time ≤90 minutes; no cessation medication or group session. If smoking cessation was part of a broader health promoting intervention, cessation had to have the mentioned content. Studies that reported results on group level-only were excluded.

### Comparison

Control participants received only brief cessation advice or information, consistent across groups.

### Outcomes

Primary outcome was biochemically validated abstinence at 12 months. Secondary outcome was self-reported abstinence at 12 months.

### Search strategy

We searched The Cochrane Central Register of Controlled Trials - CENTRAL and MEDLINE (via Ovid) from 1 January 2016 to 31 December 2023, based on search strategies shown in the Supplementary file Material 1. Articles that the search found were accepted for evaluation in any language. Grey literature was searched only for the background information of the study. Earlier studies meeting inclusion criteria were retrieved from the Cochrane review on nursing interventions for smoking cessation^9^. That study used the same search entry but without the inhaled nicotine.

### Screening of research studies and data extraction

Two authors screened the abstracts of literature search, reviewed full texts, and extracted data independently. Reasons for exclusion after both the screening and full-text review were gathered. When both authors favored inclusion after screening and full-text review, the study was qualified. Disagreements were resolved after each evaluation step through discussion.

For each trial, we extracted the following data: author(s) and year; region and country of origin; study setting and design; number and characteristics of participants and definition of ‘smoker’; description of actions in the intervention and comparison group; and biochemically validated and self-reported outcome at 12 months.

### Bias and certainty assessment

The RoB 2 tool was used to assess the risk of bias in the included studies^10^. The two authors separately assessed the risks. We did not evaluate the trials on the basis of blinding, as we tested behavioral interventions where complete blinding of participants and providers is not possible. We, however, considered if the person assessing the outcomes was unaware of which intervention group the patient is in. Robvis tool was used to present the bias analysis results^11^. Due to the small number of studies, we did not interpret the funnel plots^12^.

The review followed the Preferred Reporting Items for Systematic Reviews and Meta-Analyses (PRISMA) guidelines^13^. In accordance with PRISMA guidelines, we evaluated the methods used to assess the certainty of evidence for outcome across the included studies. Certainty of evidence was appraised in all domains of RoB 2 tool. For each outcome, we documented whether the included studies applied a formal approach to rating certainty. This evaluation was restricted to the studies included in the meta-analysis.

### Measures of treatment effect and meta-analysis

We calculated odds ratios (ORs) of cessation for individual trials and pooled results using a random-effects model in STATA 17. Where we assessed a group of studies to be clinically sufficiently homogeneous, we used the restricted maximum-likelihood method to calculate a weighted average of OR of the individual trials with 95% CI^14^. No adjustment of results was done. Leave-one-out analyses were conducted, and the results were controlled by leaving out the studies with a total high bias risk.

### Dealing with non-exact and missing data

In trials where the details of the methodology were unclear or where the results were expressed only in percentages of successful cessation, we counted out the odds ratios based on the numbers of positive and negative outcomes to get comparable numbers. Participants lost to follow-up were treated as continuing smokers.

### Assessment of heterogeneity

Heterogeneity was assessed with the I^2^ statistic^15^. I^2^ measures the percentage of total variation across studies due to heterogeneity rather than chance. Values of I^2^ over 50% indicate substantial level of heterogeneity and values over 75% considerable level of heterogeneity.

## RESULTS

### Study identification and selection

**Figure 1:**
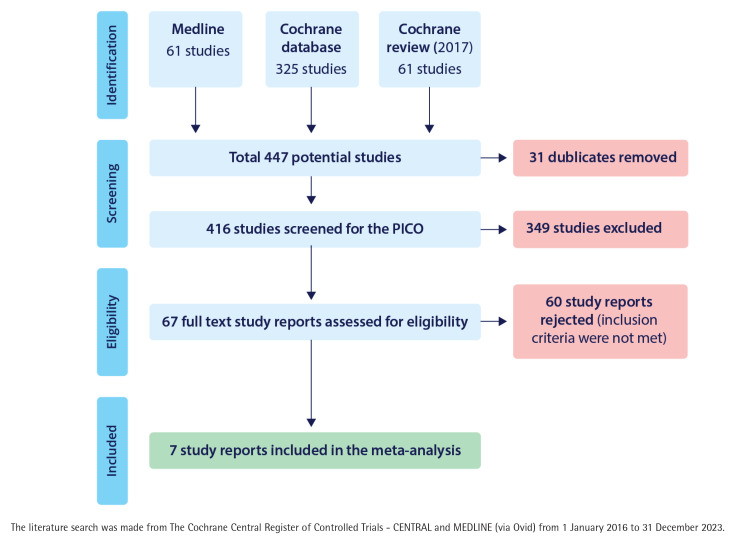
Study flow diagram for selection of articles to the meta-analysis of nurse-run smoking cessation discussions lasting up to 90 minutes

Database search gave 61 articles from MEDLINE, 325 from the Cochrane database, and 61 publications from 2017 Cochrane review. Together these gave us 447 potential studies of which 31 were duplicates. Of the rest, 349 were excluded after abstract screening (Figure 1). Of them, 96 were study plans, protocols or feasibility studies, 188 did not study smoking cessation, 30 did not study the nurses role in cessation, in 15 studies cessation medication was used, in eight studies the intervention was too intensive according to the inclusion criteria, in five studies the cessation took place by telephone only, in three studies the follow-up time was too short, two studies included people aged <18 years and two studies were not controlled trials. We considered examining the whole text of 67 articles.

### Excluded studies after full-text control

After full-text control of the 67 studies, 60 did not meet our inclusion criteria. Reasons for exclusion are described in Supplementary file Material 2. The most common reasons for exclusion were too intensive care, cessation medication used, report on group level only and cessation on telephone only without any face-to-face counselling by a nurse.

### Included studies and their characteristics

After full-text control, seven studies were accepted for the meta-analysis. All of these were conducted in hospital settings and in only one the participants represented general practice patient material^16^. Three studies were run in Hong Kong^17-19^, one in the US^20^ and three in Western Europe^16,21,22^. None of them studied electronic cigarette cessation. All together these studies included 4443 smokers.

The main characteristics of included studies are summed in Table 1 and a comprehensive description of them is in given Table 2.

**Table 1:** Summed description of included articles in the meta-analysis of nurse-run smoking cessation discussions lasting up to 90 minutes

*Details*	*Authors and Year*						
	*Chan et al.^17^* *2012*	*Curry et al.^20^* *2003*	*Hajek et al.^21^* *2002*	*Li et al.^18^* *2017*	*Li et al.^19^* *2018*	*Molto et al.^22^* *2021*	*Tønnesen et al.^16^* *1996*
**Region/Country**	Hong Kong China	Seattle USA	England UK	Hong Kong China	Hong Kong China	France	Hellerup Denmark
**Study setting**	Ten cardiac hospitals	Four pediatric clinics at hospitals	Inpatient wards in 17 hospitals	Nine hospital diabetes clinics	Five outpatient clinics of oncology departments	21 secondary and tertiary care centers	Hospital pulmonary clinic
**Design**	Single-blinded multi-center randomized controlled trial	Two-arm randomized trial	Randomized controlled trial delivered on hospital wards by cardiac rehabilitation nurses.	Randomized controlled trial to examine the effectiveness of brief intervention to promote smoking cessation and improve glycemic control in smokers with type 2 diabetes	Single-blinded simple individual randomized controlled trial	A computer-generated randomization with block design stratified per center to control the disease progress	Open, randomized study
**Number of participants**	1860	303	540	557	528	148	507
**Characteristics of participants**	Cardiac patients	Women whose children received care at pediatric clinics	Patients admitted to hospital after myocardial infarction or for cardiac bypass surgery	Patients with type 2 diabetes	Cancer patients	Axial spondylo-arthritis patients	Patients from general practice conducting chest X-ray or lung function testing
**Definition of smoker**	At least one cigarette in the past 7 days	Women who smoke cigarettes, even just sometimes	Current smokers or had recently stopped smoking	At least two cigarettes per day over the past 30 days	Smoked at least weekly in the past 6 months	Current smoker	Daily smoker
**Number of cigarettes per day**	12	12	>20 A quarter had stopped before admission	14	12–13	Not presented	Arm 1: 4 Arm 2: 17–18
**Years smoked**	39 years	18 years		38 years	42 years		
**Intervention**	30-minute individualized face-to-face smoking cessation counselling + 15-minute telephone counselling at 1 week and 1 month	Brief motivational message from the child’s clinician + self-help quide + face-to-face motivational interview with a clinic nurse + three times telephone counselling	Brief verbal advice + standard booklet + 20–30 min cessation intervention + carbon monoxide reading + special booklet + quiz + contact with other people giving up + declaration to give up	20-min face-to-face individualized counselling + DM-specific leaflet + a self-help pamphlet on quitting smoking that summarized the relationships between smoking and diabetic complications, HbA1c levels and the common misconceptions about quitting	Face-to-face individualized brief advice based on risk communication for 15–30 minutes + standard self-help smoking cessation booklet + exhaled carbon monoxide level assessment + leaflet that warns about the risks of continued smoking for subjects’ cancer treatment and prognosis + booster intervention via telephone during follow-up at 1 week	Educational program with brief verbal advice to stop smoking because it benefits the treatment of spondylo-arthritis	5 min motivational conversation (why to stop, risks of continuing) + Fagerström test for nicotine dependence + carbon monoxide level measured
**Control intervention**	15-minute face-to-face counselling on healthy diet	Usual care	Brief verbal advice + standard booklet	Glucose-oriented diabetic control + simple, brief advice + a self-help pamphlet on quitting smoking	Standard care without risk communication + standard self-help smoking cessation booklet	Comorbidly screening of spondylo-arthritis	Fagerström test for nicotine dependence + carbon monoxide level measured

The literature search was made from The Cochrane Central Register of Controlled Trials - CENTRAL and MEDLINE (via Ovid) from 1 January 2016 to 31 December 2023.

**Table 2:** Comprehensive description of the studies included in the meta-analysis

*Details*	*Description*
**Authors** **Year**	**Chan et al.^17^** **2012**
**Region/Country Study setting**	Hong Kong, China Cardiac out-patient clinics of 10 major hospitals
**Methods**	Participants were screened among all cardiac patients (60588) who visited a cardiac clinic and had smoked at least one cigarette a day (actual mean was about 12 cigarettes) during the past seven days (n=2109). After agreement 1860 patients were randomized to intervention (n=938) or control group (n=922) by opening a serially numbered sealed and opaque envelope containing printed instructions on the specified group.
**Intervention**	Stage-matched cessation counselling by a nurse counsellor (30-minute individualized face-to-face smoking cessation counselling + at 1 week and 1 month telephone follow-up) compared to a 15-minute face-to-face counselling on healthy diet by the nurse counsellor.
**Outcome**	Information (self-reported seven-day abstinence) was collected by phone at 3, 6 and 12 months by a person who was blinded to the group assignment. Non-smoking at 12 months was biochemically (exhaled carbon monoxide level <8 ppm and urine cotinine level <100 ng/mL) validated for tobacco abstinence.
**Risk of bias**	
**Randomization**	Well performed randomization. Patients in the intervention group had, however, a higher stage of readiness to quit smoking.
**Intervention**	54% received both 1-week and 1-month telephone counselling and a third of participants one of them. 10% did not receive any phone counselling.
**Missing data**	Data missing for approximately 15% of participants at 12 months. Biochemical validation performed for 29% in the intervention group and 25% in the control group.
**Measurement**	Self-reported questionnaires used for primary outcome is potentially inappropriate. In biochemical validation of quitting, the participation rates were similar.
**Selection of reporting**	Reporting followed the plan. Intention-to-treat analysis used.
**Authors** **Year**	**Curry et al.^20^** **2003**
**Region/Country Study setting**	Seattle, United States Four children care clinics serving ethnically diverse populations of low-income families
**Methods**	Mothers of children visiting the clinic screened for smoking (without defined minimum level of smoking, actual average smoking about 12 cigarettes per day) (n=303).
**Intervention**	A brief motivational message from the child’s clinician during the clinic visit, self-help guide to quitting, in-person motivational interview with a nurse and 3 outreach telephone counselling calls from the nurse compared to a brief motivational message from the child’s clinician during the clinic visit and self-help guide to quitting.
**Outcome**	Self-reported abstinence from smoking at 12 months (7-day point prevalence) and carbon monoxide exhaled (<10 ppm) validated abstinence.
**Risk of bias**	
**Randomization**	Allocation by picking a colored ball from the bag leaves some uncertainty. Intervention group was more ready to quit.
**Intervention**	Some shortages of intervention existed: face-to-face motivational interviews occurred for 74% of women and 78% received at least 1 telephone call.
**Missing data**	About 20% missing data at 12 months. Carbon monoxide testing was completed by 66%.
**Measurement**	Self-reported questionnaires used are potentially inappropriate. Intention-to-treat analyses were made.
**Selection of reporting**	Reporting followed the plan.
**Authors** **Year**	**Hajek et al.^21^** **2002**
**Region/Country Study setting**	England, UK 17 hospitals
**Methods**	Patients admitted for acute myocardial infarction or coronary by-pass operation, who smoked or had recently stopped (actual smoking some 22 cigarettes a day), were screened; 274 were allocated to intervention group and 266 to control group.
**Intervention**	Brief intervention (20–30 minutes) by a nurse including carbon monoxide reading, special booklet, quiz, contact with other quitters, commitment declaration, and sticker in notes compared to usual care (verbal advice and standard booklet).
**Outcome**	Not smoked more than five cigarettes since recruitment and self-reported point prevalence at 12 months with biochemical validation (exhaled carbon monoxide <10 ppm and salivary cotinine <20 ng/mL).
**Risk of bias**	
**Randomization**	Not explained how randomization took place.
**Intervention**	Contact with other quitters and commitment declarations took place only partially.
**Missing data**	Not clear how many patients missed the biochemical validation. They were classified as smokers.
**Measurement**	12-month abstinence was biochemically validated.
**Selection of reporting**	Self-reported point prevalence not reported. Intention-to-treat analysis was made.
**Authors** **Year**	**Li et al.^18^** **2017**
**Region/Country** **Study setting**	Hong-Kong, China Nine major diabetic clinics
**Methods**	Participants were recruited among patients with type 2 diabetes at least for 6 months and had smoked at least two cigarettes a day during the past month (mean consumption with standard deviation was 14.2 ± 9.0 cigarettes a day in the intervention group and 13.5 ± 9.2 in the control group). Of 16465 patients who were screened, 890 were eligible and 557 consented with the study. They were randomly assigned to either the intervention (283 patients) or control group (274 patients).
**Intervention**	Nurse counsellor’s 20-minute face-to-face counselling and a diabetes mellitus-specific leaflet and self-help pamphlet on quitting smoking added with two telephone contacts at one week and one month compared to usual care with a brief advice to quit smoking and self-help pamphlet on quitting.
**Outcome**	Primary outcome was 7-day point-prevalence smoking abstinence at 12 months and secondary outcomes validated 12-month quit rate and saliva cotinine [<115 ng/mL NicAlert strips (www.nymox.com)] and exhaled carbon monoxide testing (<4 ppm) for quitters at 12 months.
**Notes**	Follow-up data were collected by a person who did not know the group allocation of the patient.
**Risk of bias**	
**Randomization**	Patients in the control group had more attempts in the past to quit smoking and more physician consultations in the past 30 days.
**Intervention**	Eight percent missed the one-week and 12% one-month booster call in the intervention group and about same amount missed the follow-up in the control group.
**Missing data**	Data missing for approximately 21% of participants at 12 months.
**Measurement**	Self-reported abstinence is potentially inappropriate. Only a minority participated in biochemical validation of the result.
**Selection of reporting**	No selection in reporting was seen. Intention-to-treat analysis was used.
**Authors** **Year**	**Li et al.^19^** **2018**
**Region/Country Study setting**	Hong Kong, China Oncology department in five hospitals
**Methods**	Participants were recruited among patients who attended medical follow-up visits at oncological outpatient clinics and smoked at least weekly in the past 6 months. The mean number with standard deviation of smoked cigarettes daily was 12.5 ± 8.0 and they had smoked for 42.0 ± 13.2 years on average. Of the 43539 subjects screened, 1425 subjects were eligible. Of them, 897 showed no interest in joining the study or were unavailable for the interventions. Finally, 528 patients were randomly assigned, 268 to intervention group and 260 to the control group.
**Intervention**	Face-to-face brief advice based on risk communication + follow-up risk communication by phone delivered by a nurse counsellor compared to standard care (smoking cessation booklet).
**Outcome**	Primary outcome self-reported 7-day point prevalence abstinence at 6 months. Secondary outcomes self-reported 7-day point prevalence abstinence at 12 months and biochemically validated quit rate at 6 months. Biochemically validated quit rate at 12 months also reported [cotinine in saliva (<115 ng/mL NicAlert strips (www.nymox.com) and an exhaled carbon monoxide test <4 ppm].
**Risk of bias**	
**Randomization**	The groups differed in the level of education. Primary school or less of education was more common in the intervention group.
**Intervention**	About 8% of patients in the intervention group were lost to the follow-up risk communication by phone at one week.
**Missing data**	About 30% of patients were lost to follow-up at six months and about 40% at 12 months in both groups.
**Measurement**	Self-reported questionnaires used for primary outcome are potentially inappropriate.
**Selection of reporting**	Analyses followed the study plan and were performed by intention-to-treat method.
**Authors** **Year**	**Molto et al.^22^** **2021**
**Region/Country Study setting**	France 21 secondary and tertiary care centers
**Methods**	Participants were axial spondylo-arthritis (disease duration over a year and stable disease) patients who were recruited to a nurse-led program of self-assessment and self-management of their disease (which is negatively affected by smoking).
**Intervention**	Disease education in the intervention group (n=250) and comorbidity screening/management in the control group (n=252) by nurses. The intervention group had an educational video that was discussed with the nurse. The comparison group did not receive any educational material. In the intervention group the harmful effects of smoking were explained to patients and they were advised to stop smoking. It was possible that patients also in the comparison group were encouraged to stop smoking due to their comorbidities like heart disease.
**Outcome**	The main outcome was coping with the disease, and secondary outcomes were changes on other patient-reported outcomes, patient acceptable symptoms, disease-activity, successful smoking cessation, non-steroidal anti-inflammatory drug intake, home-based exercises and supervised physiotherapy sessions.
**Notes**	There were 74 smokers in both groups who all completed the study. The amount of smoking was not described but active smoking was mentioned.
**Risk of bias**	
**Randomization**	Computer-generated list, stratified per center was generated by an independent statistician.
**Intervention**	Completed as planned.
**Missing data**	After one year follow-up, 232 (92.8%) and 239 (94.8%) patients completed the study in the education and control groups, respectively. Among active smokers at baseline, all completed the study.
**Measurement**	The cessation was self-reported which is not appropriate.
**Selection of reporting**	No
**Authors** **Year**	**Tønnesen et al.^16^** **1996**
**Region/Country**	Denmark
**Study setting**	Hospital lung examination units
**Methods**	Participants were screened among patients that had been referred to chest radiography and/or lung function testing (n=2140). Patients smoking <10 cigarettes per day and patients smoking ≥10 cigarettes per day who refused nicotine replacement therapy were invited to participate in the study.
**Intervention**	Motivational approach (n=254) consisting of carbon monoxide measurement a 5-minute conversation, brochures, advice, and a follow-up letter 4–6 weeks later that encouraged to stop smoking compared to carbon monoxide measurement without other care (n=253).
**Outcome**	Sustained success rate was defined as subjects who stopped at initial intervention and stayed non-smokers for 12 months. Point prevalence at 12 months. Both biochemically validated (exhaled carbon monoxide <10 ppm) for quitters.
**Notes**	Intention to treat analysis used. Lost to follow-up were considered smokers.
**Risk of bias**	
**Randomization**	Not described
**Intervention**	Performed as described
**Missing data**	Twelve percent were lost to follow-up
**Measurement**	Carbon monoxide test for validation.
**Selection of reporting**	Those lost to follow-up were counted as smokers.

The literature search was made from The Cochrane Central Register of Controlled Trials - CENTRAL and MEDLINE (via Ovid) from 1 January 2016 to 31 December 2023.

### Risk of bias in included studies

**Figure 2:**
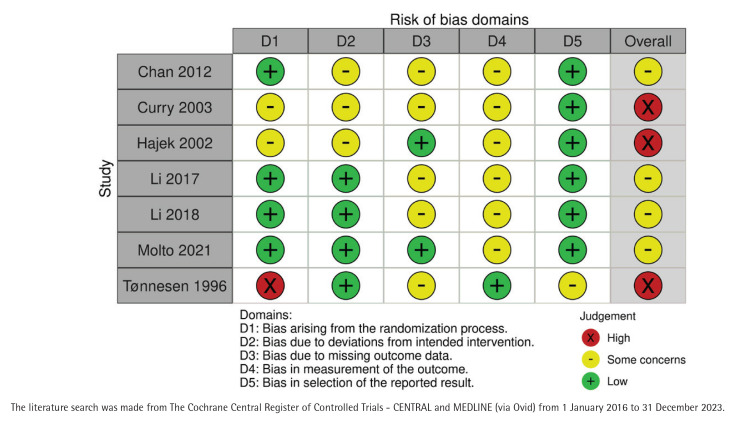
Risk of bias in the studies selected for meta-analysis of nurse-run smoking cessation discussions lasting up to 90 minutes

All included studies had some bias risks and three of them were considered to have a high total risk of bias^16,20,21^ (Figure 2). The written judgements of each domain in bias analysis are given in Table 2 and visually in Supplementary file Material 3. The earliest study did not have any description on randomization^16^. No selection of the reported results was found in any of the studies.

### Effect of intervention

**Figure 3:**
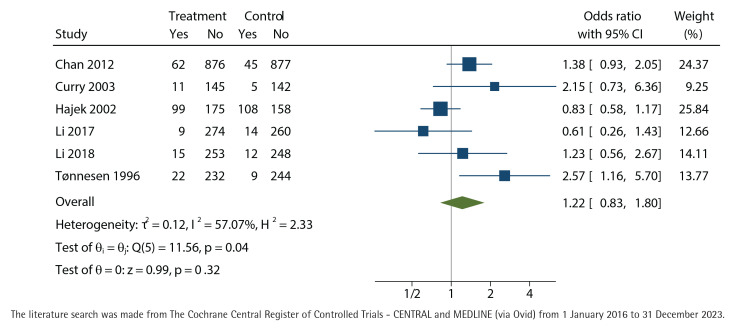
Biochemically validated unadjusted odds-ratio with 95% confidence interval (CI) of cessation at twelve months of nurse-run smoking cessation discussions lasting up to 90 minutes compared with control group without cessation discussions analyzed with the restricted maximum-likelihood method

**Figure 4:**
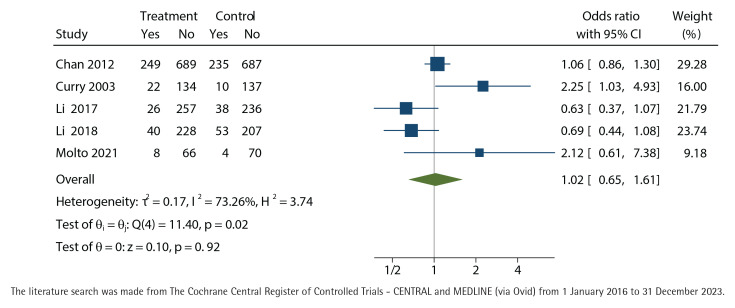
Self-reported point prevalence of cessation, unadjusted odds-ratio with 95% confidence interval (CI) at twelve months of nurse-run smoking cessation discussions lasting up to 90 minutes compared with control group without cessation discussions analyzed with the restricted maximum-likelihood method

We did not find any effect of nurse-run cessation discussions lasting up to 90 minutes, neither in the primary outcome with biochemical validation at twelve months (OR=1.22; 95% CI: 0.83–1.80) (Figure 3) nor in the self-reported outcome of point-prevalence in quitting smoking at twelve months (OR=1.02; 95% CI: 0.65–1.61) (Figure 4). Both results favored the control group but did not reach statistical significance. The results were controlled by leaving out the studies with high total bias risk, but the outcomes did not change.

### Heterogeneity of studies

Both analyses showed substantial level of heterogeneity: I^2^ was 57% for the biochemically validated studies and 73% for the self-reported studies.

## DISCUSSION

This systematic review and meta-analysis found no evidence that nurse-run smoking cessation discussions up to 90 minutes improve quit rates at 12 months. These findings contrast with a prior review demonstrating the effectiveness of nurse-run interventions^9^. In the prior meta-analysis, there were no limitations of cessation time, and it included intensive intervention studies.

One explanation why smoking cessation was not successful may be that participants in most included studies were long-term smokers with serious smoking-related illnesses, making cessation particularly difficult. Additionally, the exclusion of studies involving cessation medication may have limited observed effectiveness. Combining behavioral support with pharmacotherapy is known to increase quit rates^23^.

Five of the included seven studies in this meta-analysis were designed solely for smoking cessation^16,17,19-21^, one study for improvement of type 2 diabetes care and smoking cessation^18^ and one study for comprehensive guidance (including non-smoking) in prevention of further progress in axial spondylo-arthritis^22^. In five studies the target group had a severe disease^17-19,21,22^, in one study the target group was low-income women visiting a pediatric clinic with a child^20^, and one study included patients from general practice who were to be tested for problems in the lungs^16^. Severe diseases and difficult life situations maybe disturbed professional counselling and cessation of smokers in several included studies, as has been shown earlier^24,25^.

We used the 90-minute limitation in cessation intensity because we believe that in most countries there are limitations for nurses for how much time they can use in cessation discussions. While, according to our results, this kind of intervention did not improve cessation, it is advisable to recommend brief advice to quit smoking. A brief intervention by a physician has been shown to be effective in at least six-month follow-up^26^. This model should be further tested if it had similar results when in use by nurses. On the other hand, nurses could increase the total cessation discussion time if the operational environment allows for more extensive use of time for cessation discussions. Alternative approaches may also be warranted. Group-based counselling has demonstrated effectiveness and cost-efficiency^27^. Nurse-run interventions that incorporate promotion of pharmacotherapy may also be more effective. A Cochrane meta-analysis that studied the combined effect of pharmacotherapy and behavioral intervention in smoking cessation found a positive effect^23^. The result calls for cessation medication to be promoted even in nurse-run cessation discussions.

### Strengths and limitations

A strength of our study was that we included seven studies with over 4000 smokers in the meta-analysis that limited the total cessation discussion time in nurse-run counselling to 90 minutes. This allowed us to conclude that the nurse-run cessation should be reorganized in many practices if the total time for cessation discussion is too short. There were several limitations in our study. First, in the included cessation studies, participants had serious diseases caused by smoking. This means that they were long-standing smokers, and years of smoking cause both physical and psychological dependence of nicotine which is difficult to combat. Second, studies that used nicotine replacement therapy or cessation medication were excluded from the meta-analysis, which resulted in exclusion of several successful cessations studies because we wanted to find out the sole effect of the nurse cessation counselling. Third, the included studies had considerable risk of bias, but this did not alter the results. Fourth, we searched grey literature only for the background information when the study protocol was made, but we did not systematically search this kind of information for the meta-analysis.

Many studies on nurse-run smoking cessation are already decades old. It would be important to conduct RCTs on different methods of nurse-run tobacco and electronic cigarette cessation even today. Also, future studies should consider the cessation counselling time use. We would need studies that test the effect of a short recommendation to quit, and studies that limit nurses’ time use so that it is possible to implement an effective treatment model in real-life practice.

## CONCLUSIONS

Nurse-run smoking cessation discussions, up to 90 minutes, did not improve abstinence rates at 12 months. This should be considered while organizing cessation care; smokers that are interested in using cessation medication should be given more time and offered the possibility to participate in cessation group counselling. In other situations, only brief advice to quit. Future RCTs should focus on populations with shorter smoking histories and fewer comorbidities.

## Supplementary Material



## Data Availability

Data sharing is not applicable to this article as no new data were created.
