# Supplementary material for: Effectiveness of nurse-run smoking cessation discussions lasting up to 90 minutes: A systematic review and meta-analysis
Source: Tob Prev Cessat. 2026 Feb 21;12:10.18332/tpc/215042. doi: 10.18332/tpc/215042 (PMC12941095; doi:10.18332/tpc/215042)
Supplement: Supplementary file 1 [file TPC-12-12-s1.pdf]

Supplement 1. The search strategies

The search strategies were based on Cochrane Tobacco Addiction Group's (TAG) Specialised Register strategies (https://tobacco.cochrane.org/resources/cochrane-tag-specialised-register) with minor modifications.

1. The Cochrane Central Register of Controlled Trials (CENTRAL) search strategy:

1. SMOKING*:KW
2. TOBACCO-USE-DISORDER*:KW
3. TOBACCO-SMOKELESS*:KW
4. TOBACCO-SMOKE-POLLUTION*:KW
5. TOBACCO-USE-CESSATION*:KW
6. NICOTINE*:KW
7. (SMOKING and CESSATION):TI,AB,KW
8. ANTISMOK*:TI,AB,KW
9. QUIT*:TI
10. SMOK*:TI
11. CIGAR*:TI
12. TOBACCO:TI
13. NICOTINE:TI
14. #1 or #2 or #3 or #4 or #5 or #6 or #7 or #8 or #9 or #10 or #11 or #12 or #13
15. SR-TOBACCO
16. (#14 not #15) with Publication Year from 2016 to 2023, in Trials
17. (nurse* or nursing or health-visitor*):ti,ab,kw
18. #16 and #17
19. (electronic-cigar* or vaping or e-cigar*):ti,kw
20. #18 not #19

2. MEDLINE (via ovid) Search Strategy:

1 Randomized Controlled Trial.pt.
2 Controlled Clinical Trial.pt.
3 Pragmatic Clinical Trial.pt.
4 Equivalence Trial.pt.
5 Adaptive Clinical Trial.pt.
6 Clinical Trial.pt.
7 Meta analysis.pt.
8 exp Clinical Trial/
9 Random-Allocation/
10 randomized-controlled trials/
11 double-blind-method/
12 single-blind-method/
13 placebos/
14 Research-Design/
15 ((clin$ adj5 trial$) or placebo$ or random$).ti,ab.
16 ((singl$ or doubl$ or trebl$ or tripl$) adj5 (blind$ or mask$)).ti,ab.

17 (volunteer$ or prospectiv$).ti,ab.
18 exp Follow-Up-Studies/
19 exp Retrospective-Studies/
20 exp Prospective-Studies/
21 exp Evaluation-Studies/ or Program-Evaluation.mp.
22 exp Cross-Sectional-Studies/
23 Comparative study/
24 exp Behavior-therapy/
25 exp Health-Promotion/
26 exp Community-Health-Services/
27 exp Health-Behavior/ or exp Health-Education/
28 1 or 2 or 3 or 4 or 5 or 6 or 7 or 8 or 9 or 10 or 11 or 12 or 13 or 14 or 15 or 16 or 17 or 18 or 19 or 20 or 21 or 22 or 23 or 24 or 25 or 26 or 27
29 smoking cessation.mp. or exp Smoking Cessation/
30 tobacco cessation.mp. or "Tobacco-Use-Cessation"/
31 exp Smoking/th [Therapy]
32 "Tobacco-Use-Disorder"/
33 Tobacco-Smokeless/
34 exp Tobacco-Smoke-Pollution/
35 Smoking reduction/ or Smoking reduction.mp.
36 Smoking prevention/
37 Vaping/ or vaping.mp.
38 Electronic Nicotine Delivery Systems/
39 electronic cigar*.mp.
40 exp Pipe smoking/ or exp Tobacco smoking/ or exp Tobacco Products/
41 ((quit$ or stop$ or ceas$ or giv$ or abstain* or abstinen*) adj5 (smoking or smoke* or tobacco)).ti,ab.
42 exp Tobacco/ or exp Nicotine/
43 29 or 30 or 31 or 32 or 33 or 34 or 35 or 36 or 37 or 38 or 39 or 40 or 41 or 42
44 exp Smoking/ not 43
45 1 or 2 or 3 or 4 or 5
46 43 and 28
47 43 and 45
48 (animals not humans).sh.
49 ((29 or 30 or 32 or 33) and REVIEW.pt.) not 46
50 44 and 28
51 (50 and 45) not 48
52 46 not 47 not 48
53 (43 and 45) not 48
54 limit 53 to yr="2016 - 2023"
55 37 or 38 or 39
56 54 not 55
57 exp Nurses/
58 exp Nursing/
59 (nurse* or nursing or health visitor*).ti,ab,kw.
60 57 or 58 or 59
61 56 and 60

Supplement 2. Reasons for exclusion from the meta-analysis after full text control.

**First author and year**	**Reason for exclusion from meta-analysis**
Allen JK, 1996	Smoking prevalence was reported only on group level, so it was not possible to know who stopped, continued or started smoking during the intervention
Aveyard P, 2003	Our interest was in the nurse intervention. Participation in the nurse intervention arm was very poor, so it was not possible to study the nurses' role in cessation.
Balmumcu A, 2021	Only congress report found.
Berndt N, 2014	NRT used.
Bolman C, 2002	Intervention started with a cardiologist advice to stop smoking. This did not take place in the control group. While we were interested about the influence of nurse advice, the cardologists' advice would interfer the results.
Borrelli B, 2005	The control group with standard care got too intensive cessation support.
Brouwer-Goossensen D, 2022	Even the control group patients received two times 15-minute health behaviour advice from a nurse
Campbell NC, 1998	Cessation was part of lifestyle intervention. Smoking at baseline and outcome were reported on group level, so it was not possible to know who stopped, continued or started smoking during the intervention
Canga N, 2000	Intervention included use of NRT.
Carlsson R, 1997	Lifestyle change program. The numbers of smokers were reported in a way that it was not possible to count numbers for cessation.
Castello LM, 2022	The intervention could include also services of tobacco cessation clinic.
Cheng EM, 2018	Cessation was part of lifestyle intervention. Smoking at baseline and outcome were reported on group level, so it was not possible to find out who stopped, continued or started smoking during the intervention
Chouinard MC, 2005	The intervention was too intensive compared to our PICO and cessation medication use was allowed in the study.
Cossette S, 2012	The intervention included only telephone counselling.
Daumit GL, 2020	The intervention was run by community health coach and nurse. It was too intensive to fit our PICO.
van Dijk DJ, 2017	Too short follow-up time.
Duffy SA, 2006	Too intensive, medication used and cessation discussion only on phone.
Family Heart Study Group, 1994	Due to the study protocol the cessation outcomes could not be reported according to the intention to treat analysis.
Feeney GF, 2001	Too intensive intervention according to our PICO.
Gilbody S, 2015	NRT was used in the study.
Hansen TA, 2007	The intervention included telephone counselling only.
Hennrikus DJ, 2005	The intervention was too intensive with up to seven counselling sessions, and NRT use was allowed
Hollis JF, 1993	None of the intervention arms followed the intervention we were looking for in our PICO.
Hornnes N, 2014	Cessation intervention too intensive compared to our protocol.
Huang YJ, 2017	Too intensive intervention compared to our protocol.
Janz NK, 1987	General practitioners participated in the intervention group but not in control group.
Jiang X, 2007	Too short follow-up time.
Jorstad HT, 2013	Tobacco cessation outcomes not reported separately.
Kadda O, 2015	Too intensive intervention compared to our PICO.
Kazemzadeh Z, 2016	Too short follow-up time.
Lancaster T, 1999	There was no control group for the brief intervention and NRT use was allowed
Lu CC, 2019	Too intensive intervention compared to our PICO.
Merzaai B, 2021	Numbers of successful cessation not reported.
Meysman M, 2010	Too short follow-up time.

Miller NH,1997	Nicotine replacement therapy offered.
Navarro E, 2021	Cessation discussions took place on the phone only.
O'Donoghue B, 2022	Too intensive intervention compared to our PICO.
Olaiya MT, 2017	Cessation was part of lifestyle intervention. General practitioner participated in implementation of lifestyle change and cessation care plan. Smoking at baseline and outcome were reported on group level, so it was not possible to find out who stopped, continued or started smoking during the intervention
OXCHECK Study Group, 1994	Cessation was part of lifestyle intervention. Smoking at baseline and outcome were reported on group level, so it was not possible to find out who stopped, continued or started smoking during the intervention
Pardavila-Belio MI, 2015	The total time used for cessation intervention was too long.
Premkumar S, 2022	Short follow-up period, only 3 months.
Quist-Paulsen P, 2003	Too many nurse cessation contacts compared to our PICO.
Rice V.H, 1994	Intervention more intensive than intended in our PICO.
Rigotti NA, 1994	The study included patients who had quit smoking during the past half a year.
Risser NL, 1990	The control group had too intensive cessation intervention which even resembled the intervention in our PICO.
Sanders D, 1989	Only 25.9% in the intervention group received the intended nurse counselling, which was the main criterium in our PICO.
Sanz-Pozo B, 2006	Too intensive intervention with 6 counselling sessions
Sivarajan Froelicher ES, 2004	NRT or bupropion was used in many cases.
Smit ES, 2016	It was possible that smokers in the usual care group received intensive cessation treatment.
Smith PM, 2009	Both the intervention and control group received more smoking cessation than our PICO allowed.
Snaterse M, 2019	Intervention was over 90 min that exceeds our PICO. Depending on patients' preferences, pharmacological therapy for smoking cessation could be prescribed.
Steptoe A, 1999	Counselling was supported by NRT when appropriate
Taylor CB, 1990	Article not found.
Terazawa T, 2001	Article only in Japanese.
Towfighi A, 2021	Cessation was part of lifestyle intervention. Smoking at baseline and outcome were reported on group level, so it was not possible to find out who stopped, continued or started smoking during the intervention
Vanharen Y, 2023	The study did not follow individuals' changes in smoking and the difference from baseline to the end was not reported.
Vetter NJ, 1990	General practitioners participated in the intervention as initiators of cessation only in the intervention group.
Wan LH, 2016	Intervention took place on phone only. Results were presented for the whole group, not individuals.
Wei Z, 2021	The intervention was too intensive compared with our PICO.
Wood DA, 2008	The number of smokers presented only on group level, so it was not possible to know how many actually stopped.

NRT= nicotine replacement therapy

PICO= population, intervention, comparison, outcome

References of Supplement 2

1. Allen JK. Coronary risk factor modification in women after coronary artery bypass surgery. Nurs Res. 1996;45(5):260-265. doi:10.1097/00006199-199609000-00002.
2. Aveyard P, Griffin C, Lawrence T, Cheng K. A controlled trial of an expert system and self-help manual intervention based on the stages of change versus standard self-help materials in smoking cessation. Addiction. 2003;98(3):345-354. doi:10.1046/j.1360-0443.2003.00302.x
3. Balmumcu A, Ünsal Atan Ş. Smoking Cessation Programs for Pregnant Women: Utilizing WhatsApp Text Messaging. J Addict Nurs. 2021;32(3):188-196. doi:10.1097/JAN.0000000000000417.
4. Berndt N, Bolman C, Froelicher ES, et al. Effectiveness of a telephone delivered and a face-to-face delivered counseling intervention for smoking cessation in patients with coronary heart disease: a 6-month follow-up. J Behav Med. 2014;37(4):709-724. doi:10.1007/s10865-013-9522-9.
5. Bolman C, de Vries H, van Breukelen G. A minimal-contact intervention for cardiac inpatients: long-term effects on smoking cessation. Prev Med. 2002;35(2):181-192. doi:10.1006/pmed.2002.1036.
6. Borrelli B, Novak S, Hecht J, Emmons K, Papandonatos G, Abrams D. Home health care nurses as a new channel for smoking cessation treatment: outcomes from project CARES (Community-nurse Assisted Research and Education on Smoking). Prev Med. 2005;41(5-6):815-21. doi:10.1016/j.ypmed.2005.08.004.
7. Brouwer-Goossensen D, Scheele M, van Genugten L, et al. Motivational interviewing in a nurse-led outpatient clinic to support lifestyle behaviour change after admission to a stroke unit: a randomized controlled trial. Eur J Cardiovasc Nurs. 2022;21(1):36-45. doi:10.1093/eurjcn/zvab001.
8. Campbell NC, Ritchie LD, Thain J, Deans H, Rawles J, Squair J. Secondary prevention in coronary heart disease: a randomised trial of nurse led clinics in primary care. Heart. 1998;80(5):447-452.
9. Canga N, De Irala J, Vara E, Duaso M, Ferrer A, Martínez-González M. Intervention study for smoking cessation in diabetic patients: a randomized controlled trial in both clinical and primary care settings. Diabetes Care 2000;23(10):1455-1460. doi:10.2337/diacare.23.10.1455.
10. Carlsson R, Lindberg G, Westin L, Israelsson B. Influence of coronary nursing management follow up on lifestyle after acute myocardial infarction. Heart. 1997;77(3):256-259. doi:10.1136/hrt.77.3.256
11. Castello LM, Airoldi C, Baldrighi M, et al. Effectiveness and feasibility of smoking counselling: a randomized controlled trial in an Italian emergency department. Eur J Public Health. 2022;32(1):119-125. doi:10.1093/eurpub/ckab114.
12. Cheng EM, Cunningham WE, Towfighi A, et al. Efficacy of a Chronic Care-Based Intervention on Secondary Stroke Prevention Among Vulnerable Stroke Survivors: A Randomized Controlled Trial. Circ Cardiovasc Qual Outcomes. 2018;11(1):e003228. doi:10.1161/CIRCOUTCOMES.116.003228.
13. Chouinard MC, Robichaud-Ekstrand S. The effectiveness of a nursing inpatient smoking cessation program in individuals with cardiovascular disease. Nurs Res. 2005;54(4):243-254. doi:10.1097/00006199-200507000-00006.

14. Cossette S, Frasure-Smith N, Robert M, et al. A pilot randomized trial of a smoking cessation nursing intervention in cardiac patients after hospital discharge. Can J Cardiovasc Nurs. 2012;22(4):16-26. PMID: 23488362.
15. Daumit GL, Dalcin AT, Dickerson FB, et al. Effect of a Comprehensive Cardiovascular Risk Reduction Intervention in Persons With Serious Mental Illness: A Randomized Clinical Trial. JAMA Netw Open. 2020;3(6):e207247. doi:10.1001/jamanetworkopen.2020.7247.
16. van Dijk DJ, R Crone M, van Empelen P, J Assendelft W, J Middelkoop B. Favourable outcomes of a preventive screening and counselling programme for older people in underprivileged areas in the Netherlands: The PRIMUS project. Prev Med Rep. 2017;6:258-264. doi:10.1016/j.pmedr.2017.03.013.
17. Duffy SA, Ronis DL, Valenstein M, et al. A tailored smoking, alcohol, and depression intervention for head and neck cancer patients. Cancer Epidemiology, Biomarkers Prev. 2006;15(11):2203-2208. doi:10.1158/1055-9965.EPI-05-0880.
18. Family Heart Study Group. Randomised controlled trial evaluating cardiovascular screening and intervention in general practice: principal results of British family heart study. BMJ. 1994;308(6924):313-320.
19. Feeney GF, McPherson A, Connor JP, McAlister A, Young MR, Garrahy P. Randomized controlled trial of two cigarette quit programmes in coronary care patients after acute myocardial infarction. Intern Med J. 2001;31(8):470-475. doi:10.1046/j.1445-5994.2001.00110.x.
20. Gilbody S, Peckham E, Man MS, et al. Bespoke smoking cessation for people with severe mental ill health (SCIMITAR): a pilot randomised controlled trial. Lancet Psychiatry. 2015 May;2(5):395-402. doi:10.1016/S2215-0366(15)00091-7.
21. Hanssen TA, Nordrehaug JE, Eide GE, Hanestad BR. Improving outcomes after myocardial infarction: a randomized controlled trial evaluating effects of a telephone follow-up intervention. Eur J Cardiovasc Prev Rehabil. 2007;14(3):429-437. doi:10.1097/HJR.0b013e32801da123.
22. Hennrikus DJ, Lando HA, McCarty MC, et al. The TEAM project: the effectiveness of smoking cessation interventions with hospital patients. Prev Med. 2005 Mar;40(3):249-258. doi:10.1016/j.ypmed.2004.05.030.
23. Hollis JF, Lichtenstein E, Vogt TM, Stevens VJ, Biglan A. Nurse-assisted counseling for smokers in primary care. Ann Intern Med. 1993;118(7):521-525. doi:10.7326/0003-4819-118-7-199304010-00006.
24. Hornnes N, Larsen K, Brink-Kjaer T, Boysen G. Specific antismoking advice after stroke. Dan Med J. 2014;61(4):A4816.
25. Huang YJ, Parry M, Zeng Y, Luo Y, Yang J, He GP. Examination of a Nurse-led Community-based Education and Coaching Intervention for Coronary Heart Disease High-risk Individuals in China. Asian Nurs Res (Korean Soc Nurs Sci). 2017;11(3):187-193. doi:10.1016/j.anr.2017.07.004.
26. Janz NK, Becker MH, Kirscht JP, Eraker SA, Billi J, Woolliscroft JO. Evaluation of a minimal-contact smoking cessation intervention in an outpatient setting. Am J Public Health. 1987;77(7):805-809. doi:10.2105/ajph.77.7.805.

27. Jiang X, Sit JW, Wong TK. A nurse-led cardiac rehabilitation programme improves health behaviours and cardiac physiological risk parameters: evidence from Chengdu, China. J Clin Nurs. 2007;16(10):1886-1897. doi:10.1111/j.1365-2702.2007.01838.x.
28. Jorstad HT, von Birgelen C, Alings AM, et al. Effect of a nurse-coordinated prevention programme on cardiovascular risk after an acute coronary syndrome: main results of the RESPONSE randomised trial. Heart. 2013;99(19):1421-30. doi:10.1136/heartjnl-2013-303989.
29. Kadda O, Kotanidou A, Manginas A, Stavridis G, Nanas S, Panagiotakos DB. Lifestyle intervention and one-year prognosis of patients following open heart surgery: a randomised clinical trial. J Clin Nurs. 2015;24(11-12):1611-1621. doi:10.1111/jocn.12762.
30. Kazemzadeh Z, Manzari ZS, Vaghee S, Ebrahimi M, Mazlom SR. The impact of smoking cessation training-counseling programs on success of quitting smoking in patients with acute coronary syndrome. Journal of Evidence-Based Care. 2016;6(3):67-76.
31. Lancaster T, Dobbie W, Vos K, Yudkin P, Murphy M, Fowler G. Randomised trial of nurse-assisted strategies for smoking cessation in primary care. Br J Gen Pract. 1999;49(440):191-194.
32. Lu C, Hsiao Y, Huang H, Lin JY, Huang CL. Effects of a nurse-led, stage-matched, tailored program for smoking cessation in health education centers: A prospective, randomized, controlled trial. Clin Nurs Res. 2019;28(7): 812-829.
33. Merzaai B, Tonnesen H, Rasmussen M, Lauridsen S. Perioperative alcohol and smoking cessation intervention: impact on other lifestyles. Semin Oncol Nurs. 2021;37(1):151116. doi:10.1016/j.soncn.2020.151116.
34. Meysman M, Boudrez H, Nackaerts K, Dieriks B, Indemans R, Vermeire P. Smoking cessation rates after a nurse-led inpatient smoking cessation intervention. J Smok Cessat. 2010;5(1):69-76.
35. Miller NH, Smith PM, DeBusk RF, Sobel DS, Taylor CB. Smoking cessation in hospitalized patients. Results of a randomized trial. Arch Intern Med. 1997;157(4):409-415.
36. Navarro Correal E, Casellas Jorda F, Borruel Sainz N, et al. Effectiveness of a Telephone-Based Motivational Intervention for Smoking Cessation in Patients With Crohn Disease: A Randomized, Open-Label, Controlled Clinical Trial. Gastroenterol Nurs. 2021;44(6):418-425. doi:10.1097/SGA.0000000000000572.
37. O'Donoghue B, Mifsud N, Castagnini E, et al. A single-blind, randomised controlled trial of a physical health nurse intervention to prevent weight gain and metabolic complications in first-episode psychosis: the Physical Health Assistance in Early Psychosis (PHAstER) study. BJPsych Open. 2022;8(6):e189. doi:10.1192/bjo.2022.590.
38. Olaiya MT, Cadilhac DA, Kim J, et al. Community-Based Intervention to Improve Cardiometabolic Targets in Patients With Stroke: A Randomized Controlled Trial. Stroke. 2017;48(9):2504-2510. doi:10.1161/STROKEAHA.117.017499.
39. Imperial Cancer Research Fund OXCHECK Study Group. Effectiveness of health checks conducted by nurses in primary care: final results of the OXCHECK study. BMJ. 1995;310(6987):1099-1104.

40. Pardavila-Belio MI, Garcia-Vivar C, Pimenta AM, Canga-Armayor A, Pueyo-Garrigues S, Canga-Armayor. Intervention study for smoking cessation in Spanish college students: Pragmatic randomized controlled trial. Addiction. 2015;110(10):1676-1683. doi: 10.1111/add.13009.
41. Premkumar S, Ramamoorthy L, Pillai AA. Impact of nurse-led cardiac rehabilitation on patient's behavioral and physiological parameters after a coronary intervention: A pilot randomized controlled trial. J Family Community Med. 2022;29(1):17-23. doi:10.4103/jfcm.jfcm_315_21.
42. Quist-Paulsen P, Gallefoss F. Randomised controlled trial of smoking cessation intervention after admission for coronary heart disease. BMJ. 2003;327(7426):1254-1257.
43. Rice VH, Fox DH, Lepczyk M, et al. A comparison of nursing interventions for smoking cessation in adults with cardiovascular health problems. Heart Lung. 1994;23(6):473-486.
44. Rigotti NA, McKool KM, Shiffman S. Predictors of smoking cessation after coronary artery bypass graft surgery. Results of a randomized trial with 5-year follow-up. Ann Intern Med. 1994;120(4):287-293. doi:10.7326/0003-4819-120-4-199402150-00005.
45. Risser NL, Belcher DW. Adding spirometry, carbon monoxide, and pulmonary symptom results to smoking cessation counseling: a randomized trial. J Gen Intern Med. 1990;5(1):16-22. doi:10.1007/BF02602303.
46. Sanders D, Fowler G, Mant D, Fuller A, Jones L, Marzillier J. Randomized controlled trial of anti-smoking advice by nurses in general practice. J R Coll Gen Pract. 1989;39(324):273-276.
47. Sanz-Pozo B, Miguel-Díaz J, Aragón-Blanco M, González González AI, Cortes Catalán M, Vázquez I. Effectiveness of a programme of intensive tobacco counselling by nursing professionals [Efectividad de un programa de consejo antitabaco intensivo realizado por profesionales de enfermería]. Atencion Primaria. 2006;37:266-272.
48. Sivarajan Froelicher ES, Miller NH, Christopherson DJ, et al. High rates of sustained smoking cessation in women hospitalized with cardiovascular disease: the Women's Initiative for Nonsmoking (WINS). Circulation. 2004;109(5):587-593. doi:10.1161/01.CIR.0000115310.36419.9E
49. Smit ES, Candel MJ, Hoving C, de Vries H. Results of the PAS study: a randomized controlled trial evaluating the effectiveness of a web-based multiple tailored smoking cessation program combined with tailored counseling by practice nurses. J Health Commun. 2016;31(9):1165-1173. doi:10.1080/10410236.2015.1049727.
50. Smith PM, Burgess E. Smoking cessation initiated during hospital stay for patients with coronary artery disease: a randomized controlled trial. CMAJ. 2009;180(13):1297-1303. doi:10.1503/cmaj.080862.
51. Snaterse M, Jorstad HT, Minneboo M, et al. Smoking cessation after nurse-coordinated referral to a comprehensive lifestyle programme in patients with coronary artery disease: a substudy of the RESPONSE-2 trial. Eur J Cardiovasc Nurs. 2019;18(2):113-121. doi:10.1177/1474515118795722.
52. Steptoe A, Doherty S, Rink E, Kerry S, Kendrick T, Hilton S.. Behavioural counselling in general practice for the promotion of healthy behaviour among adults at increased risk of coronary heart disease: randomised trial. BMJ. 1999;319(7215):943-947. doi:10.1136/bmj.319.7215.943.

53. Taylor CB, Houston-Miller N, Killen JD, DeBusk RF. Smoking cessation after acute myocardial infarction: effects of a nurse-managed intervention. Ann Intern Med. 1990;113(2):118-123. doi:10.7326/0003-4819-113-2-118.
54. Terazawa T, Mamiya T, Masui S, Nakamura M. [The effect of smoking cessation counseling at health checkup]. Sangyo Eiseigaku Zasshi. 2001;43(6):207-213. In Japanese. doi:10.1539/sangyoeisei.kj00002552512.
55. Towfighi A, Cheng EM, Ayala-Rivera M, et al. Secondary Stroke Prevention by Uniting Community and Chronic Care Model Teams Early to End Disparities (SUCCEED) Investigators. Effect of a Coordinated Community and Chronic Care Model Team Intervention vs Usual Care on Systolic Blood Pressure in Patients With Stroke or Transient Ischemic Attack: The SUCCEED Randomized Clinical Trial. JAMA Netw Open. 2021;4(2):e2036227. doi:10.1001/jamanetworkopen.2020.36227.
56. Vanharen Y, Abugattas de Torres JP, Adriaenssens B, et al. Nurse-led care after ablation of atrial fibrillation: a randomized controlled trial. Eur J Prev Cardiol. 2023;30(15):1599-1607. doi:10.1093/eurjpc/zwad117.
57. Vetter NJ, Ford D. Smoking prevention among people aged 60 and over: a randomized controlled trial. Age and Ageing. 1990;19(3):164-168.
58. Wan LH, Zhang XP, Mo MM, et al. Effectiveness of Goal-Setting Telephone Follow-Up on Health Behaviors of Patients with Ischemic Stroke: A Randomized Controlled Trial. J Stroke Cerebrovasc Dis. 2016;25(9):2259-2270. doi:10.1016/j.jstrokecerebrovasdis.2016.05.010.
59. Wei Z, Zhang Y. Application of Motivational Interview Combined with Staged Nursing in Treatment of Coronary Heart Disease Patients. This article was originally published in a special issue, “Diagnostic and Therapeutic Advances in Biomedical Research and Pharmaceutical Sciences”. Indian J Pharm Sci. 2021;83(5)Spl Issue:237-242.
60. Wood DA, Kotseva K, Connolly S, et al; EUROACTION Study Group. Nurse-coordinated multidisciplinary, family-based cardiovascular disease prevention programme (EUROACTION) for patients with coronary heart disease and asymptomatic individuals at high risk of cardiovascular disease: a paired, cluster-randomised controlled trial. Lancet. 2008;371(9629):1999-2012. doi:10.1016/S0140-6736(08)60868-5.

Supplement 3. Comprehensive bias analysis including all domain questions showing the grade of concern (low, some or high). Abbreviations used: Y=Yes, PY=Partly yes, E=No information, N=No, PN=Partly no, 0=Not assessed due to the previous answers.

	**First author**	**Chan** [14]	**Curry** [17]	**Hajek** [18]	**Li** [15]	**Li** [16]	**Molto** [19]	**Tønnesen** [13]
	**Year**	2012	2003	2002	2017	2018	2021	1996
**Dom**	**Study design**	RCT	RCT	RCT	RCT	RCT	RCT	RCT
**1**	**Randomization process**							
1.1.	allocation sequence random?	PY	PY	E	Y	Y	Y	E
1.2.	allocation sequence concealed?	PY	PY	E	PY	Y	PY	E
1.3.	differences between groups?	N	PN	N	N	PN	N	N
	Risk of bias judgement DOMAIN 1	LOW	SOME	SOME	LOW	LOW	LOW	HIGH
**2**	**Deviations from the intended (assignm)**							
2.1.	participants aware of their assigned intervention?	Y	Y	Y	Y	Y	Y	Y
2.2.	carers aware of participants assigned intervention?	Y	Y	Y	Y	Y	Y	Y
2.3.	deviations of the intended intervention?	N	N	PN	N	N	N	N
2.4.	deviations affected the outcome?	0	0	PN	0	0	0	0
2.5.	deviations balanced between the groups?	0	0	PN	0	0	0	0
2.6.	was analysis appropriate (e.g. ITT)?	Y	Y	N	Y	Y	Y?	PY
2.7.	failure to analyse in the randomized group?	0	0	N	0	0	0	0
	Risk of bias judgement DOMAIN 2	SOME	SOME	SOME	LOW	LOW	LOW	LOW
**3**	**Missing outcome data**							
3.1.	data available for (nearly) all participants?	PN	N	PY	N	N	Y	N
3.2.	result was not biased by missing data?	E	E	0	E	E	0	E
3.3.	could missingness depend on its true value?	Y	Y	0	Y	Y	0	Y
3.4.	likely, that missingness depended on its true value?	PN	PN	0	PN	PN	0	PN
	Risk on bias judgement DOMAIN 3	SOME	SOME	LOW	SOME	SOME	LOW	SOME
**4**	**Measurement of the outcome**							
4.1.	method of measuring the outcome inappropriate?	PN	PN	PN	PN	PN	PN	PN
4.2.	measurement of outcome different between groups?	N	N	N	N	N	N	PN
4.3.	assessors aware of the participants' intervention?	E	E	E	N	E	E	E
4.4.	assessment influenced by knowledge of intervention?	N	PY	PY	0	PY	PY	PY
4.5.	likely that assessment influenced by knowledge of intervention?	N	PN	PN	0	PN	PN	PN
	Risk on bias judgement DOMAIN 4	SOME	SOME	SOME	SOME	SOME	SOME	SOME
**5**	**Selection of the reported results**							
5.1.	analysed with unblinded data and prespecified analysis plan?	Y	Y	Y	Y	Y	Y	Y
5.2.	numerical result selected from multiple outcome measurements?	N	N	N	N	N	N	N
5.3.	numerical result selected from multiple analyses of the data?	N	N	N	N	N	N	N
	Risk on bias judgement DOMAIN 5	LOW	LOW	LOW	LOW	LOW	LOW	LOW
**Sum**	**Overall risk of bias**	SOME	HIGH	HIGH	SOME	SOME	SOME	HIGH

© **2026 Winell K. and Ahonen J.E.**
